# Supplementary material for: The impact of national suicide prevention strategies on suicide rates in the Region of the Americas: An interrupted time-series analysis using WHO Global Health Estimates
Source: Glob Ment Health (Camb). 2026 Mar 6;13:e57. doi: 10.1017/gmh.2026.10173 (PMC13112316; doi:10.1017/gmh.2026.10173)
Supplement: Lange et al. supplementary material [file S2054425126101733sup001.docx]

**Supplementary Material**


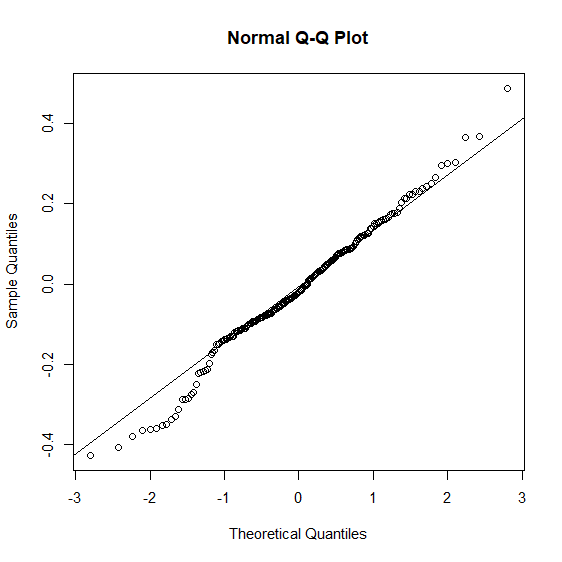


**Figure S1.** Q-Q plot for male model


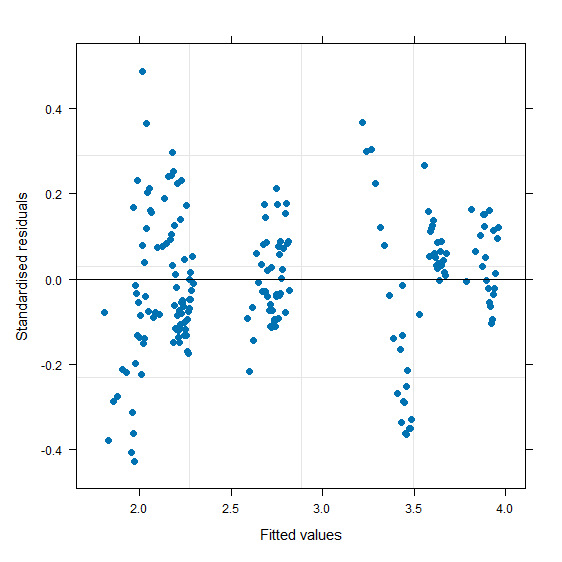


**Figure S2.** Residual vs. fitted plots for male model


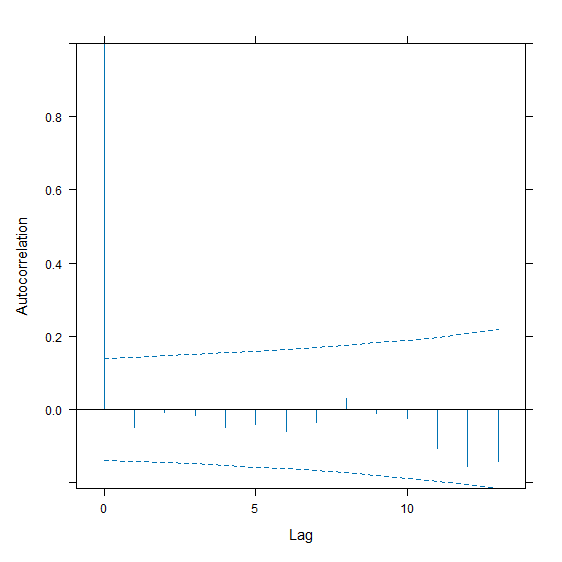


**Figure S3.** Autocorrelation plot for male model


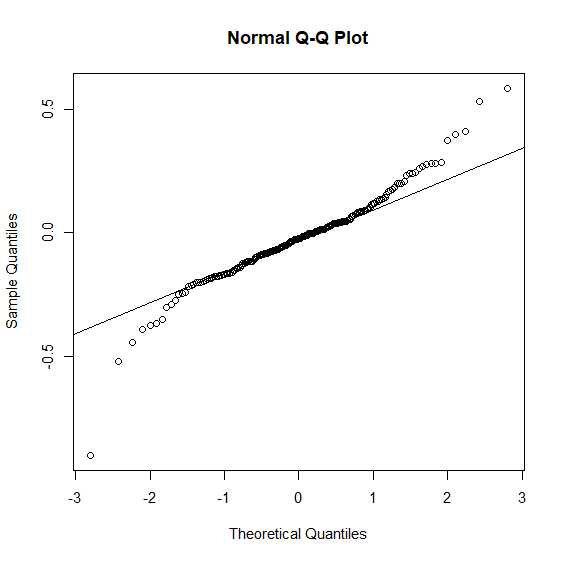


**Figure S4.** Q-Q plot for female model


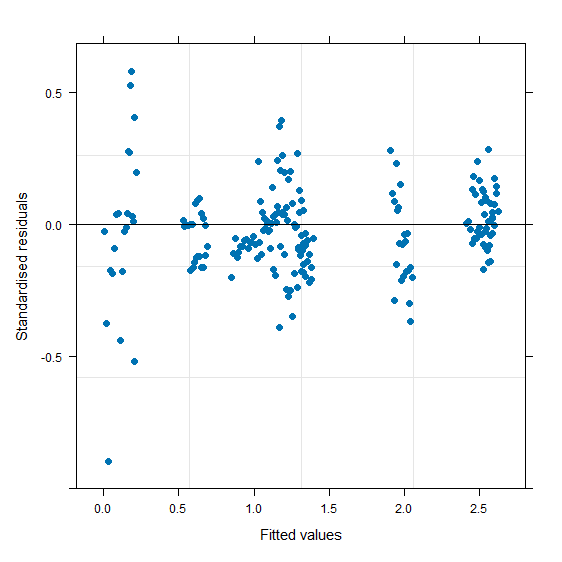


**Figure S5.** Residual vs. fitted plots for female model


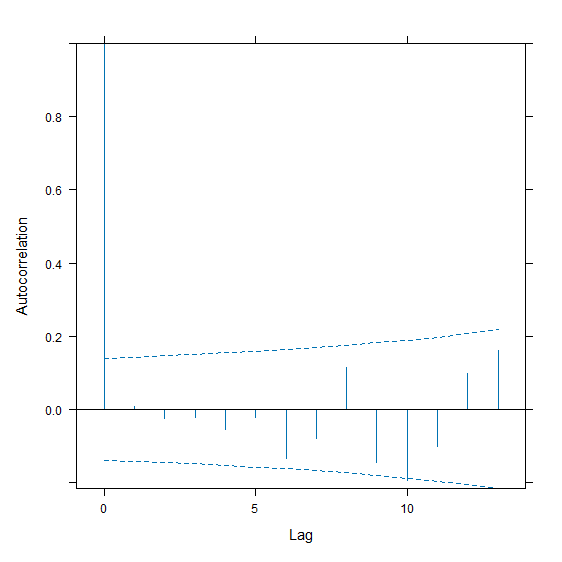


**Figure S6.** Autocorrelation plot for female model


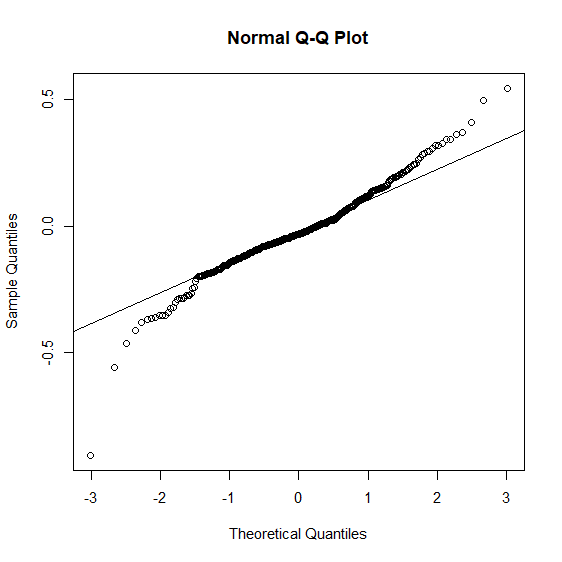


**Figure 7.** Q-Q plot for interaction model


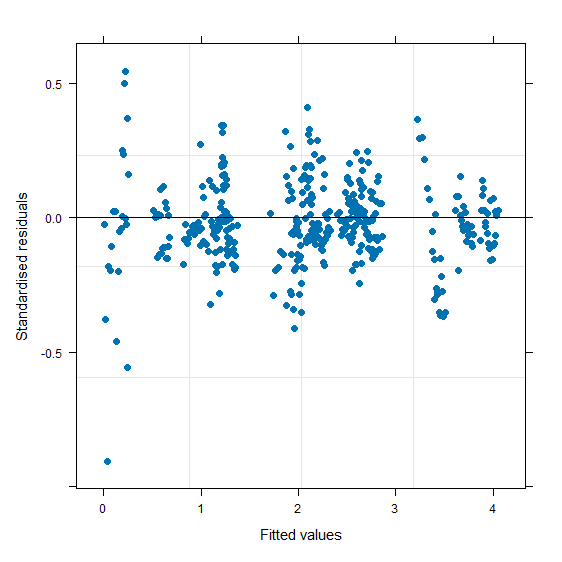


**Figure S8.** Residual vs. fitted plots for interaction model


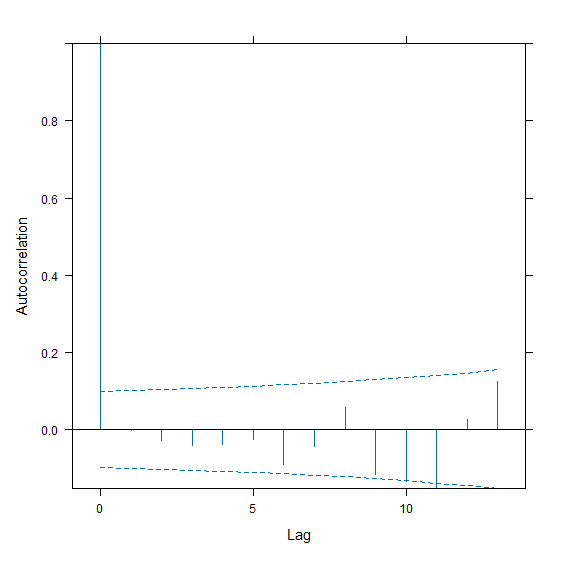


**Figure S9.** Autocorrelation plot for interaction model

**National suicide prevention strategy resources**

**Table S1.** National suicide prevention strategy resources by country

| **Country, year first implemented** | **Resource** |
| --- | --- |
| Argentina, 2015 | Prevención del Suicidio, Ley 27.130 (Suicide Prevention, Law 27.130)  <https://www.argentina.gob.ar/justicia/derechofacil/leysimple/salud/prevencion-del-suicidio>  Ley Nacional de Prevención del Suicidio, Decreto 603/2021 (National Law on Suicide Prevention, Decree 603/2021)  <https://www.argentina.gob.ar/normativa/nacional/353991/texto> |
| Brazil, 2019 | Agenda de Ações Estratégicas para a Vigilância e Prevenção do Suicídio e Promoção da Saúde no Brasil (Strategic Action Agenda for Suicide Surveillance and Prevention and Health Promotion in Brazil)  <https://www.gov.br/saude/pt-br/centrais-de-conteudo/publicacoes/cartilhas/2017/17-0522-cartilha-agenda-estrategica-publicada-pdf/view>  Lei nº 13.819, de 26 de Abril de 2019 (Law no 13,819 of 26 April 2019)  <https://www.gov.br/saude/pt-br/acesso-a-informacao/participacao-social/conselhos-e-orgaos-colegiados/cgpnpas/atos-normativos/lei-no-13-819-de-26-de-abril-de-2019.pdf/view> |
| Chile, 2013 | Programa Nacional de Prevencion del Suicidio (National Suicide Prevention Program)  <https://uchile.cl/dam/jcr:a952b085-6686-4a7b-81e4-c015bccf18ab/programa-nacional-prevencion.pdf>  <https://www.dipres.gob.cl/597/articles-212538_doc_pdf1.pdf> |
| Dominican Republic, 2014 | Programa de prevención de la conducta suicida en la República Dominicana (Prevention program in suicidal behavior the Dominican Republic)  <https://extranet.who.int/mindbank/item/6094> |
| Guyana, 2015 | National Suicide Prevention Plan 2015-2020  <https://extranet.who.int/mindbank/item/6321>  National Mental Health Action Plan and National Suicide Prevention Plan for Guyana 2024-2030  <https://health.gov.gy/wp-content/uploads/2024/04/Guyana-National-Mental-Health-and-Suicide-Plan-Final.pdf> Ley Nº 6169 / De Prevención Y Atención de Personas En Riesgo de Suicidio (Law No. 6169 / On the Prevention and Care of People at Risk of Suicide) <https://www.bacn.gov.py/leyes-paraguayas/8689/ley-n-6169-de-prevencion-y-atencion-de-personas-en-riesgo-de-suicidio> |
| Panama, 2006 | Plan para la prevencion y control de la conducta suicida en Panama 2006 (National plan for prevention of suicide 2006)  <https://www.mindbank.info/item/6093>  Ley 174 Que establece elm arco jurídico del abordaje integrap de las conductas de Riesgo suicida (Law 174 Establishing the legal framework for the comprehensive approach to suicidal risk behaviors)  <https://s3-legispan.asamblea.gob.pa/legispan/NORMAS/2020/2020/LEY/Administrador%20Legispan_29147_2020_11_2_ASAMBLEA%20NACIONAL_174.pdf>  <https://www.gacetaoficial.gob.pa/pdfTemp/29147/81655.pdf> |
| Paraguay, 2018 | Ley N° 6.169 de prevención y atención de personas en riesgo de suicidio  (Law No. 6.169 prevention and care of people at risk of suicide)  <https://www.bacn.gov.py/leyes-paraguayas/8689/ley-n-6169-de-prevencion-y-atencion-de-personas-en-riesgo-de-suicidio> |
| Suriname, 2016 | Nationaal suicide preventie – en interventieplan, 2016-2020 (National suicide prevention – an intervention plan, 2016-2020)  <https://www.mindbank.info/item/6765> |
| Uruguay, 2011 | Plan Nacional de Prevención del Suicidio, Para Uruguay 2011-2015 “Un compromiso con la vida” (National Suicide Prevention Plan for Uruguay 2011-2015. “A commitment to life.”)  [https://www.asse.com.uy/aucdocumento.aspx?10652,76651__;!!FxkXuJIC!YRVAgfbd_8G0By8saGpRTErWhA1arfa1DlTmC5X6YkiQMrsBFLYrxUzMCmrRzeT3yochsAXmwvM8uPLIXhsrfvuaYDUHKw$](https://urldefense.com/v3/__https://www.asse.com.uy/aucdocumento.aspx?10652,76651__;!!FxkXuJIC!YRVAgfbd_8G0By8saGpRTErWhA1arfa1DlTmC5X6YkiQMrsBFLYrxUzMCmrRzeT3yochsAXmwvM8uPLIXhsrfvuaYDUHKw$) |
